# Effects of β‐sitosterol on anxiety in migraine‐induced rats: The role of oxidative/nitrosative stress and mitochondrial function

**DOI:** 10.1111/cns.14892

**Published:** 2024-09-20

**Authors:** Ali Vafaei, Ahmad Vafaeian, Arad Iranmehr, Ehsan Nassireslami, Behnam Hasannezhad, Yasaman Hosseini

**Affiliations:** ^1^ Toxicology Research Center AJA University of Medical Sciences Tehran Iran; ^2^ Tehran University of Medical Sciences Tehran Iran; ^3^ Neurosurgery Department, Sina Hospital Tehran University of Medical Sciences Tehran Iran; ^4^ Gammaknife Center, Yas Hospital Tehran University of Medical Sciences Tehran Iran; ^5^ Cognitive and Behavioral Research Center AJA University of Medical Sciences Tehran Iran

**Keywords:** anxiety, frontal cortex, migraine, mitochondria, nitrosative stress, oxidative stress

## Abstract

**Aims:**

Anxiety often coexists with migraine, and both conditions share a commonality in oxidative/nitrosative stress and mitochondrial dysfunction contributing to their pathogenesis. β‐Sitosterol, a plant sterol, has shown promise in mitigating oxidative/nitrosative stress, enhancing mitochondrial function, and exerting neuroprotective effects. In this study, we investigated the impact of β‐sitosterol on migraine‐associated anxiety and whether this effect was associated with alleviation of oxidative/nitrosative stress and improvement in mitochondrial function.

**Methods:**

Nitroglycerin was used to induce migraine in adult male Wistar rats. β‐Sitosterol treatment consisted of daily intraperitoneal injections (10 mg/kg) for 10 days following migraine induction. Anxiety levels were evaluated using open‐field test (OFT) and hole‐board test (HBT). Frontal cortex samples were analyzed for malondialdehyde (MDA), glutathione (GSH), reactive oxygen/nitrogen species, nitric oxide (NO) (markers of oxidative/nitrosative stress), and ATP (indicator of mitochondrial function).

**Results:**

Migraine induction led to impaired performance in both the OFT and the HBT. Concurrently, it elevated MDA, reactive oxygen/nitrogen species, and NO levels while diminishing GSH levels in the frontal cortex, signifying heightened oxidative/nitrosative stress. Moreover, ATP levels decreased, indicating mitochondrial dysfunction. Treatment with β‐sitosterol significantly restored performance in both behavioral assays and normalized the levels of MDA, GSH, reactive oxygen/nitrogen species, NO, and ATP.

**Conclusion:**

β‐Sitosterol exerted anxiolytic effects in migraine, which can be attributed to its ability to ameliorate oxidative/nitrosative stress and enhance mitochondrial function.

## Graphical abstract

Anxiety often coexists with migraine, with both conditions sharing commonalities in oxidative/nitrosative stress and mitochondrial dysfunction contributing to their pathogenesis. In this study, we found β‐sitosterol to reduce anxiety in migraine‐induced rats, likely via its antioxidant and cellular metabolism enhancing effects.

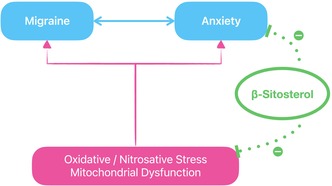

## 1 INTRODUCTION

Migraine is characterized by recurrent episodes of intense headaches accompanied by symptoms like nausea and heightened sensitivity to light, sound, and motion. Migraines can be triggered by a variety of factors, including dietary choices, physical activity, weather changes, and more. Migraine primarily affects individuals under the age of 50, resulting in substantial disability and societal burdens.^1^ Worldwide, around 15% of the population experience migraine, making it the third most common disorder on a global scale.^2^ Notably, migraine stands as a leading contributor to disability.^3^ Anxiety disorders are prevalent among migraine patients, and these two conditions often co‐occur.^4^ The interplay between migraine and anxiety is intricate and well‐established, encompassing various biological, psychological, and environmental factors.^5^ The relationship appears to be bidirectional, with frequent migraine headaches leading to higher anxiety levels and vice versa.^6^

The underlying pathophysiology of both migraine and anxiety is subject to ongoing research. Emerging evidence suggests that exacerbated oxidative/nitrosative stress and impaired mitochondrial function in various brain regions contribute to the pathogenesis of both conditions.^7^, ^8^, ^9^ Oxidative stress occurs when there is an imbalance between the production of reactive oxygen species (ROS) and the body's ability to neutralize or repair the damage caused by these molecules. ROS include molecules like superoxide anion, hydrogen peroxide, and hydroxyl radicals, which are natural byproducts of cellular metabolism. Under normal circumstances, the body has antioxidant systems, such as enzymes and molecules like glutathione (GSH), to counteract the harmful effects of ROS.^10^ Nitrosative stress is closely related to and often occurs in conjunction with oxidative stress. Nitrosative stress occurs due to an imbalance between the production of reactive nitrogen species (RNS) and the body's ability to manage them. RNS, such as nitric oxide (NO) and peroxynitrite (ONOO‐), are involved in various cellular signaling pathways and have both beneficial and detrimental effects depending on their concentrations.^11^ Mitochondrial dysfunction refers to a condition where mitochondria fail to function correctly, leading to a decline in ATP production, the accumulation of damaged mitochondrial DNA, and increased ROS production.^12^ The interconnectedness of these concepts lies in the fact that oxidative/nitrosative stress contributes to mitochondrial dysfunction. When ROS and RNS levels are elevated, they can damage mitochondrial components, including mitochondrial DNA, proteins, and lipids. This damage impairs mitochondrial function and leads to a vicious cycle of increased ROS and RNS production, further exacerbating oxidative/nitrosative stress.^10^, ^11^, ^12^

β‐Sitosterol, a phytosterol, is a steroidal compound commonly found in plant foods, with the highest concentrations typically found in vegetable oils. In plants, its primary role is to stabilize the phospholipid bilayer of the cell membrane. Functionally and structurally, it shares similarities with cholesterol in animals. β‐Sitosterol is frequently used as a dietary supplement and has been designated as generally recognized as safe (GRAS) by the Food and Drug Administration (FDA).^13^ Notably, β‐sitosterol possesses the capacity to penetrate the blood–brain barrier (BBB) and accumulate within the brain.^14^ There is a well body of evidence indicating that β‐sitosterol can mitigate oxidative/nitrosative stress, enhance mitochondrial function, and demonstrate neuroprotective properties, with some supporting its positive impact on a number of neurological disorders.^13^

In this study, we investigated the impact of β‐sitosterol on anxiety associated with migraine. After establishing the beneficial effects of β‐sitosterol, we conducted an in‐depth exploration of the underlying mechanisms responsible for this effect, with a focus on oxidative/nitrosative stress and mitochondrial function.

## 2 METHODS AND MATERIALS

### 2.1 Animals

Adult 10‐week‐old male Wistar rats were employed in this study. All animals were naïve to any previous experimental procedures and were treated in accordance with the guidelines outlined in the *Guide for the Care and Use of Laboratory Animals*.^15^ The animals were housed in standard polycarbonate cages (45 cm long × 30 cm wide × 20 cm high) with stainless steel wire mesh lids and wood shavings bedding. Wood wool was provided on top of the bedding as nesting material. The cages were kept in a room exclusively dedicated to rats, maintained at a temperature of 23 ± 1°C, with a humidity of 55 ± 3%, and a 12‐h light/dark cycle (lights on at 07:00). The animals were grouped in 2–3 rats per cage and had access to standard chow and water ad libitum. Prior to the onset of experiments, all animals underwent a one‐week acclimation period. During this time, they were familiarized with the laboratory environment, which included daily transferring between holding and testing rooms, as well as handling by the experimenter. All experiments were carried out between 09:00 a.m. and 12:00 p.m. to reduce the impact of circadian hormonal fluctuations and minimize potential sources of variability.

### 2.2 Study design

Forty‐eight animals were distributed randomly into four groups, with 12 animals allocated to each. In behavioral assays, data were gathered from the entire cohort of 12 animals within each group. However, for biochemical assays, data were obtained from a randomly selected subset of 6 animals in each group. The selection of the number of animals and drug regimens were based on prior research,^16^, ^17^ as well as our own pilot studies.

- Control group: This group did not receive any injections.
- Vehicle group: Migraine was induced through a series of five injections of nitroglycerin (10 mg/kg) on Days 1, 3, 5, 7, and 9. Subsequently, daily injections of DMSO 0.5% were administered from Days 10–19.
- Sumatriptan group (positive control): Migraine was induced with the same series of nitroglycerin injections. Subsequently, daily injections of sumatriptan (1 mg/kg) were administered from Days 10–19.
- β‐Sitosterol group: Migraine was induced with the same series of nitroglycerin injections. Subsequently, daily injections of β‐sitosterol (10 mg/kg) were administered from Days 10–19.

**FIGURE 1:**
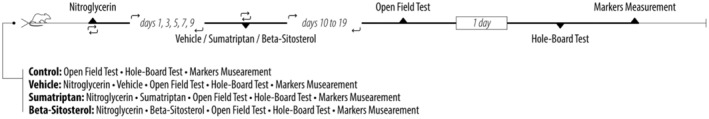
Study design. Events timeline and groups regimens. *n* = 12 for behavioral assays. *n* = 6 for markers measurement.

On Day 20, all groups underwent open‐field test (OFT), followed by hole‐board test (HBT) on Day 21. After completing the behavioral assays, the animals were euthanized, and samples of the frontal cortex were collected to measure oxidative/nitrosative stress and mitochondrial function markers (Figure 1). Experimenters were blinded to the study groups for conducting the behavioral assays and markers measurements.

### 2.3 Drugs

All drugs and solvents used in the study were ACS grade and maintained according to the manufacturer's recommendations. Nitroglycerin solution (1,466,506, Sigma‐Aldrich) was diluted in 0.9% saline. Sumatriptan (PHR2579, Sigma‐Aldrich) and β‐sitosterol (S1270, Sigma‐Aldrich) were dissolved in 0.9% saline and 0.5% DMSO, respectively. All solutions were freshly prepared on the day of the experiments. All injections were administered intraperitoneally at a volume of 10 mL/kg.

### 2.4 Migraine induction

To induce migraine, intermittent nitroglycerin dosing was employed. The rats were administered nitroglycerin injections (10 mg/kg) every other day over a 9‐day period, totaling five injections on Days 1, 3, 5, 7, and 9. This model was first described by Pradhan et al.^16^ and has consistently proven reliable in inducing migraine in rodents. The model results in both acute and chronic migraines. Acute migraine begins in about 2 h following each injection, and the chronic migraine persists up to at least 2 weeks after the final injection.^17^

### 2.5 Behavioral assays

#### 2.5.1 Open‐field test (OFT)

OFT, originally introduced by Hall et al.,^18^ is a widely employed tool in behavioral research for evaluating various behavioral facets, including anxiety. To perform the test, we utilized a white polypropylene square arena measuring 60 × 60 cm, with walls standing at a height of 40 cm. The central zone of the arena was marked with a 30 × 30 cm square. Each rat was placed on the central square, and their time spent on this area was recorded for a duration of 10 min. The entry and exit from the central zone were defined as the moment when the animal used all four paws to enter or exit the zone. A longer time spent on the central zone is indicative of less anxiety levels in this test.

#### 2.5.2 Hole‐board test (HBT)

Derived from the OFT, HBT was initially introduced by Bossier et al.^19^ and has since been widely used to evaluate multiple aspects of behavior, including anxiety. To conduct this test, we employed a white polypropylene square arena measuring 60 × 60 cm, featuring 40 cm high walls. The arena had 16 evenly spaced holes, each with a diameter of 4 cm, and was positioned at a height of 30 cm from the ground. Each rat was placed on the center of the arena, and the number of head‐dips was counted for 5 min. A head‐dip was defined as the animal placing its head into one of the holes to a minimum depth such that its ears were level with the arena. In this test, a higher number of head‐dips indicate lower anxiety levels.

Between each session, the entire OFT and HBT apparatuses were cleansed using a wet cloth and ethanol to eliminate any potential olfactory cues or residual contaminants. During the testing period, the OFT and HBT apparatuses were dimly illuminated to 100 lux of white light. We maintained a tranquil testing environment with minimal ambient noise, supplemented by the low background hum of an air conditioner to further mask any unavoidable sounds. The testing room remained devoid of other activities, and animal behavior was monitored via a camera, with the experimenter situated in an area enclosed by curtains. No animals were culled or subjected to blood collection within the facility for at least 24 h preceding the tests. All experiments were conducted within the same room and using the same OFT and HBT apparatuses.

### 2.6 Oxidative/nitrosative stress and mitochondrial function markers measurement

After euthanasia by decapitation,^20^ the frontal cortex was dissected on an ice‐cold surface. Tissue samples were rapidly frozen in liquid nitrogen and then stored at −80°C for a few days before conducting the biochemical assays. We utilized the following assay kits from Abcam for our measurements: Lipid Peroxidation (MDA) Assay Kit (ab118970), GSH Detection Assay Kit (ab65322), DCF ROS/RNS Assay Kit (ab238535), NO Assay Kit (ab65328), and ATP Assay Kit (ab83355). The levels of malondialdehyde (MDA), GSH, reactive oxygen/nitrogen species (ROS/RNS), NO, and ATP were determined following the manufacturer's instructions. MDA, NO, and ATP levels were measured using spectrophotometry, while GSH and ROS/RNS were measured using fluorometry. It is important to note that for NO, the total levels of nitrite/nitrate were measured as an indicator of NO levels.

MDA, GSH, ROS/RNS, and NO are used as markers of oxidative/nitrosative stress,^21^, ^22^, ^23^ while ATP levels provide insights into mitochondrial function.

### 2.7 Statistical analysis

After confirming normality with the Shapiro‐Wilk test and homogeneity of variance with the Brown‐Forsythe test, one‐way ANOVA with Tukey's correction for multiple comparisons was employed for further data analysis. A *p* < 0.05 was considered as indicating a significant difference. Data are expressed as mean ± standard deviation (SD). GraphPad Prism 9 software was used to statistically analyze the data.

## 3 RESULTS

### 3.1 β‐Sitosterol reduced migraine‐associated anxiety in the rats

**FIGURE 2:**
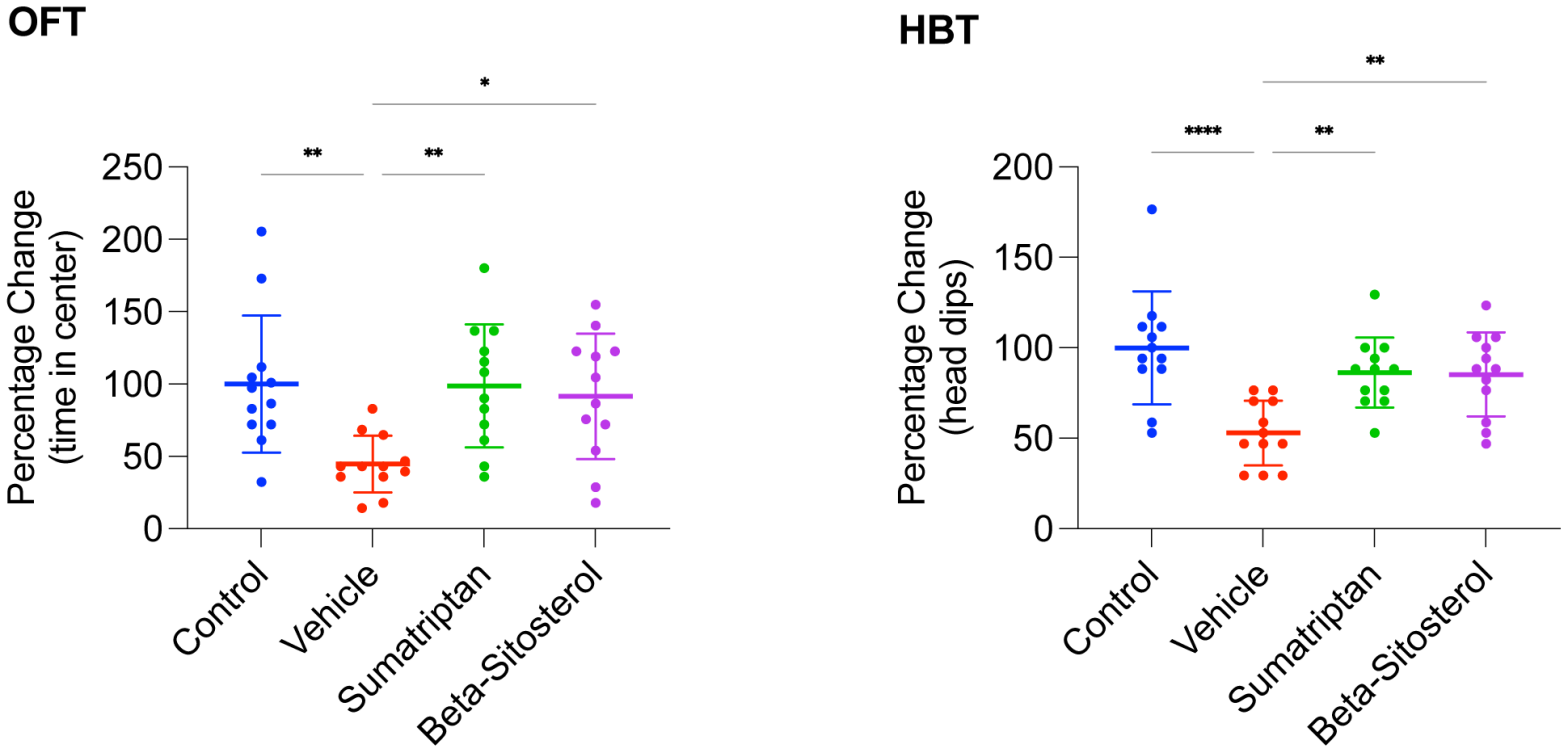
Performance in the OFT and HBT. Time spent in the central zone in OFT and number of head‐dips in HBT presented in percentage change relative to the control average. Data are presented as mean ± standard deviation. *n* = 12, for all groups; one‐way ANOVA with Tukey's correction for multiple comparisons, for all data analyses; *p < 0.05, **p < 0.01, and ****p < 0.0001.

To review the study timeline and groups, refer to Figure 1. As shown in Figure 2, the vehicle group exhibited significantly less time on the central zone of the OFT (*p* < 0.01) as well as a significantly lower number of head‐dips in the HBT (*p* < 0.0001) compared to the control group. However, both sumatriptan and β‐sitosterol groups demonstrated significantly better performance than the vehicle group in both the OFT (*p* < 0.01, *p* < 0.05, respectively) and the HBT (*p* < 0.01, for both), with results being comparable to the control group (*n* = 12, for all groups; one‐way ANOVA with Tukey's correction for multiple comparisons, for all data analyses).

In summary, induction of migraine resulted in significantly elevated anxiety levels in the rats. Furthermore, treatment with either sumatriptan or β‐sitosterol significantly restored anxiety levels, comparable to those observed in the control rats.

### 3.2 β‐Sitosterol attenuated oxidative/nitrosative stress in the frontal cortex of migraine‐induced rats

**FIGURE 3:**
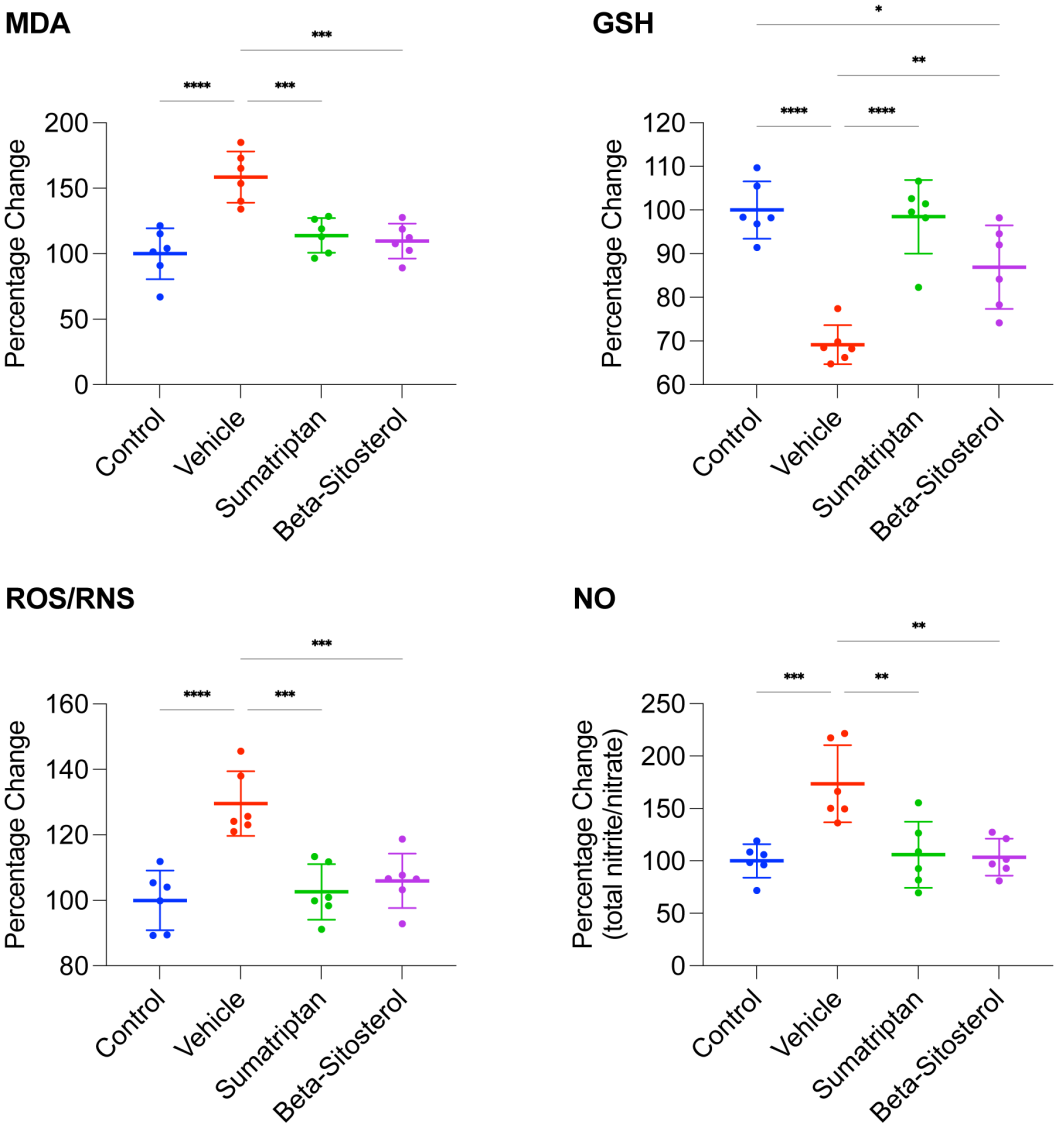
Oxidative/nitrosative stress markers levels. MDA, GSH, ROS/RNS, and NO levels presented in percentage change relative to the control average. For NO, the total levels of nitrite/nitrate were measured as an indicator of NO levels. Data are presented as mean ± standard deviation. *n* = 6, for all groups; one‐way ANOVA with Tukey's correction for multiple comparisons, for all data analyses; *p < 0.05, **p < 0.01, and ***p < 0.001 ****p < 0.0001.

The study timeline and groups are depicted in Figure 1. The findings presented in Figure 3 demonstrate that the vehicle group exhibited significantly higher levels of MDA, ROS/RNS, and NO (*p* < 0.0001, *p* < 0.0001, *p* < 0.001, respectively) and significantly lower levels of GSH (*p* < 0.0001) compared to the control group. However, the MDA, GSH, ROS/RNS, and NO levels observed in both the sumatriptan and β‐sitosterol groups were significantly restored (*p* < 0.001, *p* < 0.0001, *p* < 0.001, and *p* < 0.01, respectively, for sumatriptan group; *p* < 0.001, *p* < 0.01, *p* < 0.001, and *p* < 0.01, respectively, for β‐sitosterol group) compared to the vehicle group. Additionally, levels of MDA, ROS/RNS, and NO in sumatriptan and β‐sitosterol groups were comparable to those in the control group (*n* = 6, for all groups; one‐way ANOVA with Tukey's correction for multiple comparisons, for all data analyses).

These results implicate that migraine induction led to significant oxidative/nitrosative stress in the frontal cortex of the rats, which was significantly reduced by either sumatriptan or β‐sitosterol treatment.

### 3.3 β‐Sitosterol enhanced mitochondrial function in the frontal cortex of migraine‐induced rats

**FIGURE 4:**
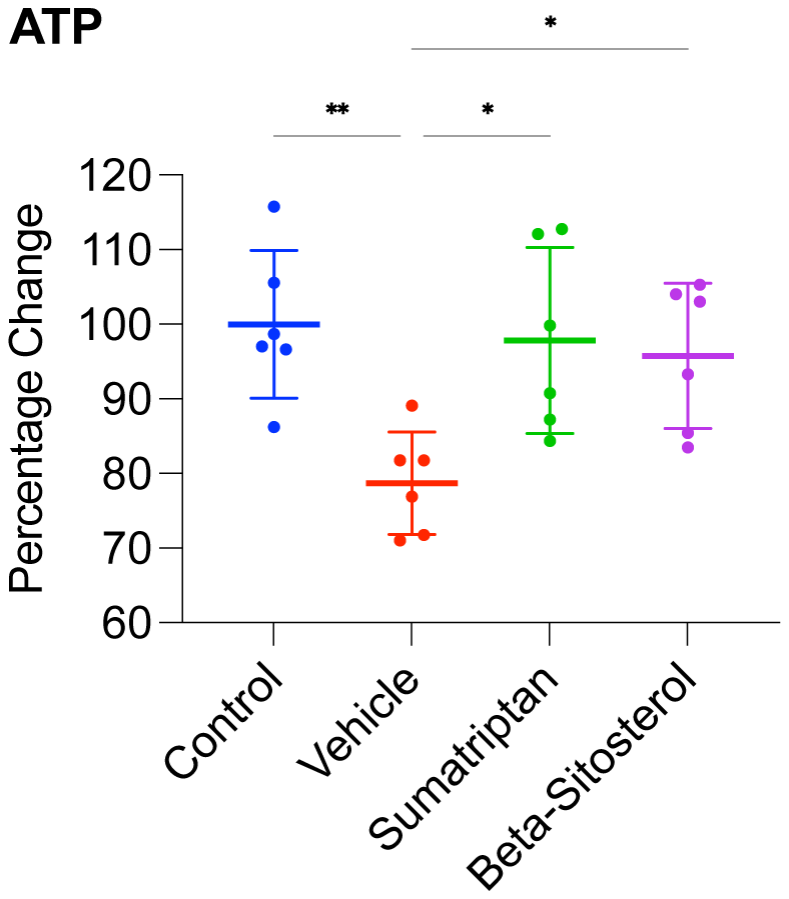
ATP levels. ATP levels, an indicator of mitochondrial function, presented in percentage change relative to the control average. Data are presented as mean ± standard deviation. *n* = 6, for all groups; one‐way ANOVA with Tukey's correction for multiple comparisons, for all data analyses; *p < 0.05, **p < 0.01.

To examine the study's timeline and groups, see Figure 1. As depicted in Figure 4, the vehicle group displayed significantly reduced ATP levels (*p* < 0.01) in comparison with the control group. Conversely, the ATP levels in both the sumatriptan and β‐sitosterol groups were significantly higher than those in the vehicle group (*p* < 0.05 for both), comparable to the levels observed in the control group (*n* = 6, for all groups; one‐way ANOVA with Tukey's correction for multiple comparisons, for all data analyses).

These findings suggest that the induction of migraine resulted in significant mitochondrial dysfunction in the frontal cortex, and treatment with either sumatriptan or β‐sitosterol restored the mitochondrial function significantly, back to levels comparable to the control rats.

## 4 DISCUSSION

In this study, we showed that β‐sitosterol treatment following nitroglycerin‐induced migraine mitigated migraine‐associated anxiety, as evidenced by improved performance in OFT and HBT. Moreover, this effect was associated with reduced levels of MDA, ROS/RNS, and NO, and increased levels of GSH in the frontal cortex, indicating attenuation of oxidative/nitrosative stress. Additionally, ATP levels were elevated in the frontal cortex, demonstrating improved mitochondrial function. Overall, our results suggest that the anxiolytic effects of β‐sitosterol in migraine may be attributed to its ability to alleviate oxidative/nitrosative stress and enhance mitochondrial function.

In line with our study's findings, numerous other studies also have highlighted the potential anti‐migraine effects of various substances that act by mitigating oxidative/nitrosative stress, regulating mitochondrial function, and modulating energy production. Magnesium, among other roles, is required for energy production, oxidative phosphorylation, and glycolysis. Magnesium deficiency is a well‐evidenced risk factor for migraine. Several double‐blind, randomized, placebo‐controlled trials have demonstrated the effectiveness of magnesium in alleviating migraine headaches. As a result, oral magnesium has been recommended for migraine pain relief in various national and international guidelines.^24^ Coenzyme Q10 (CoQ10 or ubiquinone) participates in crucial cellular redox reactions, influencing cellular metabolism and antioxidant defense. CoQ10 connects mitochondrial function with energy production and the management of oxidative stress.^25^ Several randomized controlled trials suggest that CoQ10 is a safe adjunctive treatment with evidence of efficacy in managing migraine.^26^ In a recent meta‐analysis, which reviewed six randomized controlled trials that used CoQ10 either as a standalone treatment or as an adjunct therapy, a decrease in the duration and frequency of migraine attacks was shown following CoQ10 supplementation.^27^ Lipoic acid (LA) possesses antioxidant properties, regenerates other antioxidants, and enhances mitochondrial function, including mitochondrial superoxide dismutase (SOD2) activity.^28^ A randomized, double‐blind, placebo‐controlled trial involving 44 migraine patients suggested the potential benefit of LA in migraine prevention.^29^ Another study revealed a reduction in migraine attacks in patients with insulin resistance who received LA in addition to their ongoing treatment.^30^ In a cross‐sectional study, Gross et al. observed that approximately 90% of 32 patients with high‐frequency episodic migraine had abnormally low LA levels. The authors also identified changes in markers of mitochondrial dysfunction and oxidative stress, highlighting the potential of LA as a migraine marker and reinforcing the role of mitochondrial metabolism in migraine pathogenesis.^31^ Carnitine plays a crucial role in fatty acid metabolism by aiding in their transfer to the mitochondrial matrix.^32^ Several studies have indicated a potential deficiency of carnitine in individuals experiencing migraine and have showed improvements in these conditions following carnitine supplementation.^7^ For instance, a recent case study by Charleston et al. identified carnitine deficiency in a patient with chronic migraine‐like headaches. Notably, the patient's headaches significantly improved after receiving carnitine supplementation. This suggests that carnitine deficiency should be considered in the evaluation of refractory migraines.^33^ The therapeutic potential of thiamine in migraine has a long history, with its successful use in headache treatment dating back to 1949.^34^ Mammalian cells acquire thiamine from the environment and convert it into thiamine pyrophosphate (TPP) in the cytoplasm. Most TPP is transported to mitochondria through the mitochondrial thiamine pyrophosphate transporter (MTPPT), where it plays its crucial role in energy metabolism.^35^ In two recent case studies, thiamine supplementation was reported to improve symptoms of chronic cluster headaches and chronic migraine.^36^, ^37^ Riboflavin, which is essential to energy metabolism, has shown to be effective in migraine prophylaxis in adults and adolescents.^38^, ^39^ Di Lorenzo et al. observed that riboflavin is more effective in migraine patients with non‐H mitochondrial DNA haplotypes. These results were attributed to the association of haplogroup H with increased activity in complex I, a primary target of riboflavin.^40^ Niacin, comprising nicotinic acid and nicotinamide, serves as a nutritional precursor for nicotinamide adenine dinucleotide (NAD) and nicotinamide adenine dinucleotide phosphate (NADP). These compounds play crucial roles in cellular metabolism.^41^ A large cross‐sectional study found robust association between dietary niacin intake and migraine.^42^ In a case study, sustained‐release niacin administration significantly improved the condition of a patient suffering from migraine attacks.^43^ In another study, administering a combination of low doses of niacin, tryptophan, calcium, caffeine, and acetylsalicylic acid (ASA) shortly after a migraine attack produced positive outcomes in 9 out of 12 migraine patients.^44^ Pyridoxine, folic acid, and cobalamin play roles in mitochondrial homeostasis, energy production, and antioxidant defense. Several studies have indicated the benefits of pyridoxine, folate, and cobalamin supplementation in migraine patients.^45^

Our results suggest β‐sitosterol to have neuroprotective potential through alleviating oxidative/nitrosative stress and improving mitochondrial function. Other research has also highlighted similar properties for β‐sitosterol. In one study, β‐sitosterol mitigated aluminium chloride‐mediated neurotoxicity in C57BL/6 mice. Cognitive impaired animals exhibited improved performance in Y‐maze, passive avoidance test, and novel object recognition test following β‐sitosterol treatment. β‐Sitosterol also demonstrated antioxidant effects, as indicated by increased GSH levels in the corticohippocampal brain regions of the aluminium chloride‐induced animals.^46^ In another study, β‐sitosterol was shown to reduce deficiencies in spatial learning, locomotor activity, and motor coordination caused by sodium metavanadate in mice. β‐Sitosterol also lowered MDA and hydrogen peroxide levels and increased the activity of catalase and superoxide dismutase (SOD) in the brains of vanadium‐exposed mice. Therefore, the neuroprotective effects of β‐sitosterol were associated with its ability to attenuate oxidative stress.^47^ Another study examined the effects of β‐sitosterol on a sub‐strain of transgenic mice recapitulating major features of Alzheimer's disease (AD) amyloid pathology. β‐Sitosterol‐treated transgenic animals showed improvements in spatial learning, memory, and motor coordination as evaluated by shallow water maze, Y‐maze, and balance beam test. β‐Sitosterol also reduced the free radicals load in the frontal cortex and hippocampus. Additionally, in vitro studies were conducted which reaffirmed the antioxidant effects of β‐sitosterol.^48^ In a study by Lee et al., β‐sitosterol mitigated neuronal toxicity induced by β‐Amyloid25‐35 in PC12 cells. Subsequent investigations revealed reduced levels of ROS, NO, and inducible nitric oxide synthase (iNOS) following β‐sitosterol treatment. These findings suggest that the neuroprotective effects of β‐sitosterol were exerted by inhibiting oxidative/nitrosative stress.^49^ Several other studies have also demonstrated the antioxidant activity of β‐sitosterol, with some delving deeper to further elucidate the mechanisms underlying this effect. A study involving RAW 264.7 macrophages demonstrated that β‐sitosterol prevented oxidative/nitrosative stress induced by phorbol myristate acetate (PMA), indicated by reduced superoxide anion, hydrogen peroxide, NO, and iNOS levels.^50^ In a different study using the same cell culture model, it was observed that β‐sitosterol restored GSH/total GSH ratio, while also boosting the activities of manganese superoxide dismutase (MnSOD) and glutathione peroxidase (GPx). This antioxidant effect was likely mediated through the estrogen receptor/phosphatidylinositol 3 kinase (PI3K) pathway.^51^ Consistent with this, Shi et al. showed that incorporation of β‐sitosterol into the plasma membrane of HT22 and primary hippocampal cells resulted in suppression of glucose oxidase (GOX)‐induced oxidative stress and lipid peroxidation through estrogen receptor/PI3K/glycogen synthase kinase 3 beta (GSK3β) pathway. In this study, ROS and MDA levels were used as markers for oxidative stress and lipid peroxidation measurement.^52^ In a separate study by Shi et al., introduction of β‐sitosterol into the mitochondrial membrane of HT22 cells enhanced mitochondrial function, as evidenced by increased ATP levels. This effect was attributed to an augmentation in the fluidity of inner mitochondrial membrane following β‐sitosterol integration, leading to elevated mitochondrial membrane potential (∆Ψm).^53^ In another study, β‐sitosterol exhibited protective effects in renal impaired rats by upregulating nuclear factor erythroid 2‐related factor 2 (Nrf2), a transcription factor that regulates the basal and induced expression of an array of antioxidant response element‐dependent genes.^54^

Addressing the limitations of our study is imperative. While our results underscore the anxiolytic effects of β‐sitosterol in migraine, it is crucial to recognize that these outcomes were observed in a single animal model of migraine and through two behavioral assays evaluating anxiety. Given the complexity of migraine and anxiety, both encompassing a wide range of pathologies and symptoms, we advocate for additional animal studies that explore β‐sitosterol's effects using further migraine induction and anxiety evaluation models. Furthermore, although our findings indicate that the anxiolytic effects of β‐sitosterol in migraine‐induced rats may be due to its ability to moderate oxidative/nitrosative stress and improve mitochondrial function, future research is necessary to establish a definitive causal relationship.

Future clinical trials are essential to establish the efficacy and safety of β‐sitosterol in managing migraine among human populations. We suggest the effects of β‐sitosterol to be evaluated in migraine patients as part of an enhanced combination therapy. As previously mentioned, β‐sitosterol is commonly found in plant foods and has been designated as GRAS by the FDA. Besides the combination therapy approach, the effects of β‐sitosterol in migraine patients could also be examined in the context of dietary recommendations, as part of a comprehensive migraine management strategy. Altogether, β‐sitosterol can be viewed as a potentially safe and effective adjunct to existing migraine therapies, offering prospects for improving the quality of life for those affected by migraine.

## 5 CONCLUSION

Our study suggests β‐sitosterol to have anxiolytic effects in migraine, potentially due to its antioxidant and cellular metabolism enhancing properties. This study, along with other research, paves the way toward enhanced migraine therapies through adjunctive use of β‐sitosterol, as well as other antioxidants and cellular metabolism enhancing compounds.

## AUTHOR CONTRIBUTIONS

A.V. conceptualized and designed the study, conducted the experiments, collected and analyzed the data, and prepared the manuscript. Y.H. contributed to study conceptualization and design, was involved in conducting the experiments and data collection, and supervised the entire process. A.V.N., A.I., E.N., and B.H. were also involved in conducting the experiments and data collection. All authors reviewed and approved the final manuscript.

## FUNDING INFORMATION

This study was funded by AJA University of Medical Sciences.

## CONFLICT OF INTEREST STATEMENT

The authors declare no conflicts of interest.

## ETHICS STATEMENT

This study was approved by the Ethics Committee of AJA University of Medical Sciences (Approval No.: IR.AJAUMS.REC.1401.004).

## Data Availability

All data generated or analyzed during this study are available from the corresponding author on reasonable request.
